# Single-Dose Versus Extended Antibiotic Prophylaxis in Primary Hip and Knee Arthroplasty: A Systematic Review and Meta-Analysis

**DOI:** 10.7759/cureus.74049

**Published:** 2024-11-19

**Authors:** André Fernandes, Jae Y Park, Liron Leibovitch, Iqbal F Sayudo, Elcio Machinski, Jesica Sudarman, Heather Tetley, Khalid Malik Tabassum

**Affiliations:** 1 Trauma and Orthopedics, York and Scarborough Teaching Hospitals NHS Foundation Trust, York, GBR; 2 Trauma and Orthopedics, Imperial College London, London, GBR; 3 Medicine, Azrieli Faculty of Medicine, Bar-Ilan University, Safed, ISR; 4 Medicine, Syiah Kuala University, Banda Aceh, IDN; 5 Orthopedics, State University of Ponta Grossa, Ponta Grossa, BRA; 6 Medicine, Atma Jaya Catholic University of Indonesia, Jakarta, IDN; 7 Trauma and Orthopedics, Royal Surrey County Hospital, Guildford, GBR

**Keywords:** antibiotics, arthroplasty, hip, hip replacement, knee, periprosthetic joint infection, prophylaxis, surgical site infection, systematic review, systematic review and meta analysis

## Abstract

The optimal duration of antibiotic prophylaxis for patients undergoing primary hip or knee arthroplasty remains debated. We conducted a systematic review and meta-analysis to compare the outcomes of single-dose versus extended antibiotic prophylaxis. Studies assessing these strategies for periprosthetic joint infection (PJI), revision surgery, and superficial surgical site infections were selected from systematic searches in PubMed, Embase, and the Cochrane Library. Results were synthesized using random-effects meta-analysis models. Nine studies were included, covering 295,654 patients - 125,489 undergoing total knee arthroplasty and 172,055 undergoing total hip arthroplasty. A significant statistical difference in the incidence of PJI favored single-dose over extended antibiotic prophylaxis (OR 0.78; 95% CI 0.63-0.98; I² = 0%). Our meta-analysis suggests that a single-dose prophylactic antibiotic regimen may be preferable for reducing PJI incidence in primary total hip and knee arthroplasty.

## Introduction and background

Joint arthroplasty is one of the most successful orthopedic procedures, offering pain relief, restoring function, and improving independence in a specific group of patients for whom mobility is critical [1,2]. However, the success of joint arthroplasty comes with its challenges, and one of the most crucial factors for ensuring positive outcomes is the prevention of postoperative infections. Total hip and knee arthroplasty patients require careful antibiotic prophylaxis both perioperatively and postoperatively to reduce the risk of surgical site infections (SSIs), periprosthetic joint infections (PJIs), and the need for revision surgery. Among these complications, PJI is particularly concerning due to its significant impact on patients and the healthcare system [3].

The optimal duration of prophylactic antibiotic therapy remains an ongoing topic of debate. Traditionally, a short course of antibiotics was prescribed postoperatively, but recent evidence suggests that a single dose of antibiotics administered perioperatively may provide adequate protection against infection [4].

In this meta-analysis, we pooled data from 295,654 patients across nine studies who underwent elective total hip or knee arthroplasty and compared the efficacy of single-dose versus extended antibiotic prophylaxis. Despite variability in antibiotic protocols across the studies, we were able to draw conclusions regarding the primary endpoints of interest. Our findings suggest that a single-dose prophylactic antibiotic regimen may be preferable for reducing the incidence of PJIs.

The results presented in this manuscript were initially shared as an abstract at the 44th SICOT Orthopaedic World Congress, held in Belgrade, Serbia, in September 2024.

## Review

Methods

This systematic review and meta-analysis (SRMA) was conducted and reported in strict accordance with the methodological guidelines outlined in the Cochrane Collaboration Handbook for Systematic Reviews of Interventions. Additionally, the study’s reporting structure and content were carefully aligned with the Preferred Reporting Items for Systematic Reviews and Meta-Analyses (PRISMA) statement, ensuring comprehensive and transparent documentation of the research process and findings [5].

Search Strategy

A comprehensive systematic search was conducted on Cochrane, Embase, and PubMed on July 10, 2024, and was later expanded to include Scopus, although no additional reports were identified. As shown in Appendix A, the search strategy utilized the terms "primary hip" or "hip replacement" or "hip arthroplasty" or "primary knee" or "knee replacement" or "knee arthroplasty," combined with at least one of the following terms: "single dose antibiotic" or "antibiotic prophylaxis".

Eligibility Criteria and Study Selection

This SRMA included studies that met the following eligibility criteria: (1) randomized controlled trials (RCTs) and non-RCTs; (2) studies comparing single-dose antibiotic prophylaxis to extended antibiotic prophylaxis protocols; (3) studies involving patients undergoing primary total hip arthroplasty (THA) or total knee arthroplasty (TKA); (4) studies with a follow-up of at least one year; and (5) studies reporting any relevant outcome of interest.

Exclusion criteria included (1) studies involving participants aged under 18 years; (2) studies on patients undergoing arthroplasty for trauma, revision surgery, or cancer; (3) studies where antibiotics were used for therapeutic purposes; (4) non-English articles; and (5) other article types, including animal studies, biomechanical studies, cadaveric studies, case reports, technical notes, conference abstracts, comments, editorials, and letters.

To enhance the search strategy and identify additional relevant studies, we manually reviewed the reference lists of both included studies and relevant prior systematic reviews. This approach ensured a comprehensive exploration of the literature. Two independent reviewers (AF and JYP) performed the literature search based on the predefined inclusion criteria. They independently assessed the quality of the studies and collaborated to extract relevant data, minimizing bias and ensuring the reliability and validity of the data collection process. The prospective SRMA protocol was registered on PROSPERO with the identifier #CRD42023484838 on February 15, 2024.

Data Extraction

The investigators extracted the following information from the included studies: (1) baseline characteristics of the studies and (2) primary outcomes, which included the rate of SSIs, rate of PJIs, and rate of revision surgery.

Quality Assessment

The risk of bias assessment was conducted independently by two researchers (AF and IS). Both investigators used the Risk of Bias 2 (RoB 2) tool for randomized trials and the Risk of Bias in Non-randomized Studies of Interventions (ROBINS-I) tool for non-randomized studies, following the guidelines established by the Cochrane Collaboration [6].

Data Analysis

Continuous outcomes were analyzed using mean differences, while binary outcomes were assessed through risk ratios (RR), with both measures presented alongside their respective 95% CIs. All statistical analyses were performed using Review Manager 5.4 software (The Cochrane Collaboration, London, UK). Subgroup analysis was not conducted due to insufficient data (fewer than three outcomes available) for secondary endpoints, such as the American Society of Anesthesiologists score, steroid use, diabetes mellitus, peripheral vascular disease, antibiotic timing, acute kidney injury, and MRSA status.

Assessment of Heterogeneity

Heterogeneity was assessed using Cochrane’s Q test and I² statistics. Statistical significance for heterogeneity was defined as p < 0.10 and I² > 25%. The interpretation of heterogeneity measures followed the guidelines outlined in the Cochrane Handbook for Systematic Reviews of Interventions.

Results 

Literature Search

**Figure 1:**
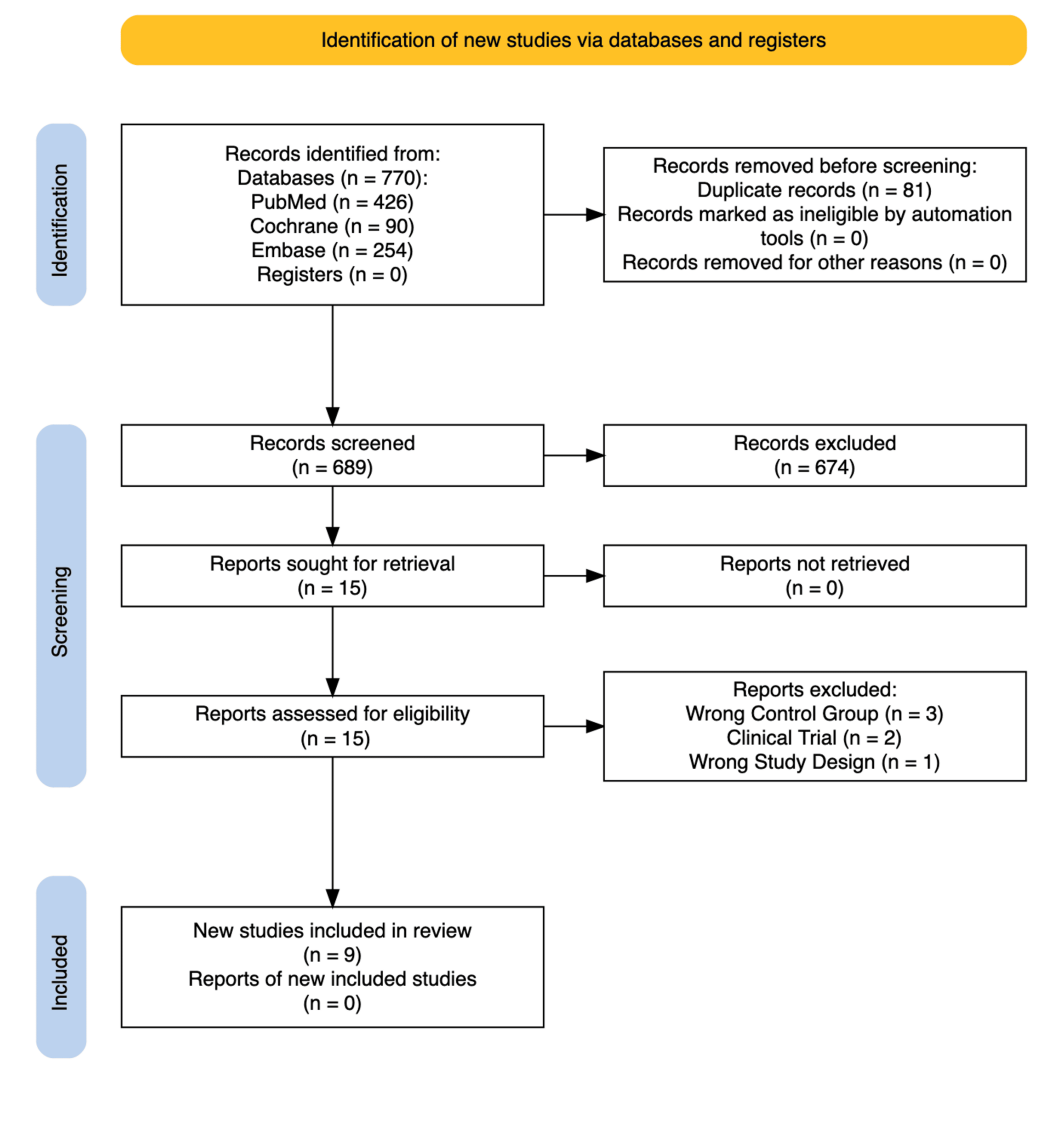
PRISMA flow diagram of study screening and selection PRISMA: Preferred Reporting Items for Systematic Reviews and Meta-Analyses

The systematic literature review process, as shown in Figure 1, identified a total of 770 articles from multiple databases: PubMed (n = 426), Cochrane (n = 90), and Embase (n = 254). After removing 81 duplicate entries, two independent reviewers (AF and JYP) screened the remaining articles. This led to the exclusion of 674 articles based on title and abstract evaluation. Subsequently, 15 papers underwent full-text review, with six excluded for non-compliance with the predefined inclusion criteria (Figure 1). The final selection for this SRMA included three RCTs and six non-RCTs [7-15].

Characteristics of the Included Studies

The baseline characteristics of the included studies are presented in Table 1. The aggregate analysis included a total of 295,654 subjects, with 125,489 TKAs (41.79%) and 172,055 THAs (58.19%). These data were derived from three RCTs and six non-RCTs comparing single-dose antibiotic prophylaxis with extended antibiotic prophylaxis protocols. The follow-up periods ranged up to 12 months post-intervention (Table 1).

**Table 1:** Baseline characteristics of included studies ^a^ Male percentage is provided for the full SP/EP cohort. ^b^ Data presented in person-years unit (see Appendix B for details). ^c^ Data is provided as a combined cohort for both SP and EP groups. EP: extended dose prophylaxis; RCT: randomized controlled trial; SP: single-dose prophylaxis; THA: total hip arthroplasty; TKA: total knee arthroplasty

Study	Design	Patients TKA SP/EP	Patients THA SP/EP	Male, SP/EP	Age, years SP/EP	Follow-up (months)	Antibiotic choice, SP/EP	Duration of antibiotic EP (days)
Christensen et al. (2021) [7]	Non-RCT	261/1,442	293/1,321	253/1,271	66.32/66.11	3	Cefazolin, vancomycin, clindamycin/cefazolin, vancomycin, and clindamycin	One day
Periti et al. (1999) [8]	RCT	75/76	347/348	129/132	66.60/64.7	12	Teicoplanin/cefazolin	One day
Tan et al. (2019) [9]	Non-RCT	1,679/7,650	2,844/8,509	2,183/720	62.3/63.3	12	Cefazolin, vancomycin/cefazolin, and vancomycin	One day
Tang et al. (2003) [10]	Non-RCT	792/125	360/90	300/60	72.3/70.8 (TKA); 55.2/52.4 (THA)	12	Cefazolin/cefuroxime	Not available
Veltman et al. (2020) [11]	Non-RCT	9,880/101,587	11,455/119,257	Appendix^b^	Appendix^b^	12	Cefazolin/cefazolin and cefuroxime	One day
Wymenga et al. (1992) [12]	RCT	0	1,327/1,324	287/266	69/69	12	Cefuroxime/cefuroxime	One day
Suter et al. (1994) [13]	RCT	0	260/260	65/75	66.5/68.2	12	Teicoplanin/cefamandole	One day
Engesaeter et al. (2003) [14]	Non-RCT	0	18,844/3,326	5,590/1,011	73.8/72.28	12	Cephalothin/cefuroxime, cloxacillin, and dicloxacillin	Not available
Van Kasteren et al. (2007) [15]	Non-RCT	0	649/1,241	596^c^	68.8^c^	12	Cefazolin, flucloxacillin, erythromycin, clindamycin/cefamandole, cefuroxime, amoxicillin, and netilmicin	Less than one day (40.6%); more than one day (22.7%)

Quality Assessment

**Figure 2:**
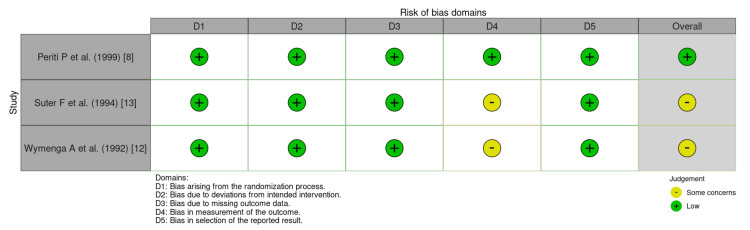
RoB 2 risk of bias assessment for RCTs D1: bias arising from the randomization process; D2: bias due to deviations from intended interventions; D3: bias due to missing outcome data; D4: bias in the measurement of the outcome; D5: bias in the selection of the reported result RCT: randomized controlled trial; RoB 2: Risk of Bias 2

The risk of bias analysis was conducted using the RoB 2 tool for RCTs and the ROBINS-I tool for non-RCTs, in accordance with Cochrane recommendations. Among the three included RCTs, Periti et al. demonstrated a very low overall risk of bias, while Suter et al. and Wymenga et al. exhibited some concerns, particularly in domain 4 (deviation from intended interventions) (Figure 2) [9,12,13].

**Figure 3:**
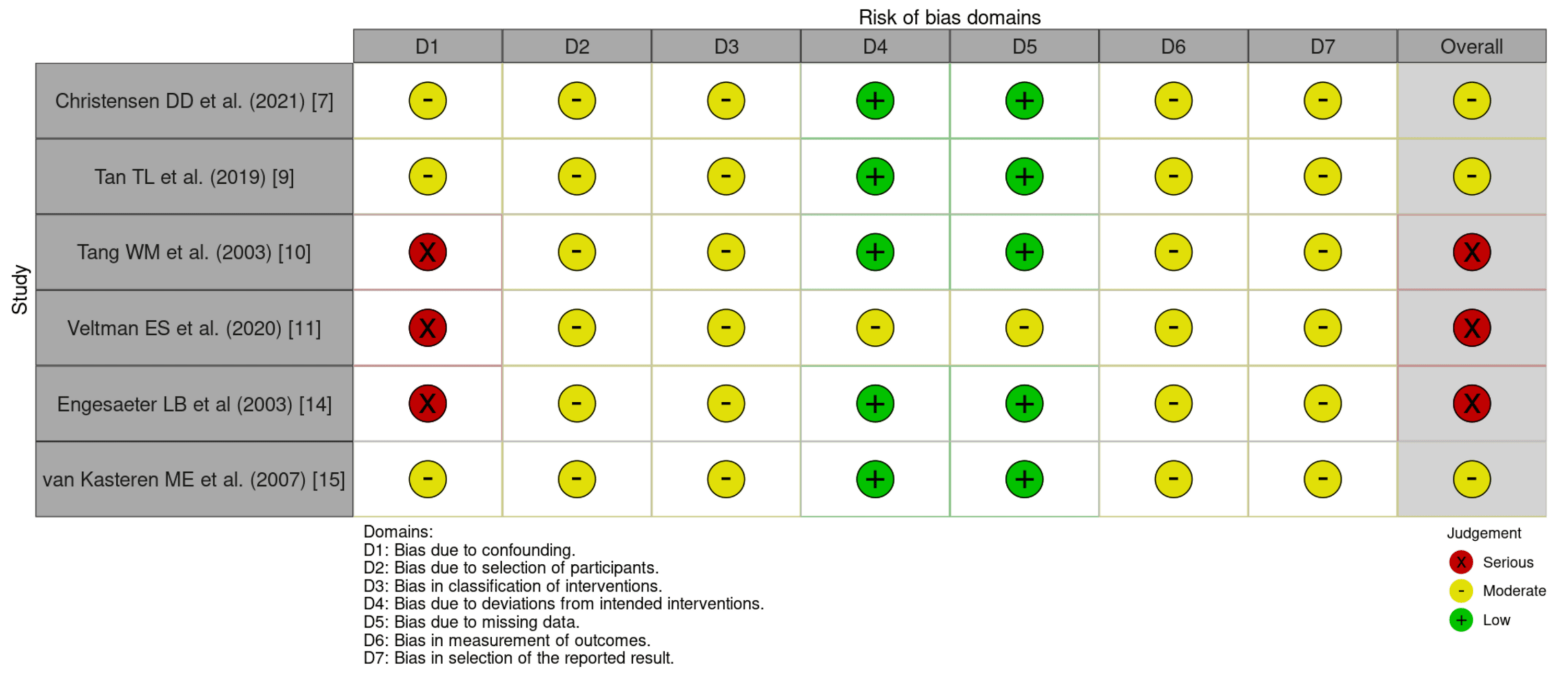
ROBINS-I risk of bias assessment for non-RCTs D1: bias due to confounding; D2: bias due to selection of participants; D3: bias in classification of interventions; D4: bias due to deviation from intended interventions; D5: bias due to missing data; D6: bias in the measurement of outcomes; D7: bias in the selection of the reported result RCT: randomized controlled trial; ROBINS-I, Risk of Bias in Non-randomized Studies of Interventions

Regarding the non-RCTs, three studies exhibited moderate concerns, while the remaining three demonstrated serious concerns regarding potential biases. Figure 3 provides a detailed risk of bias analysis for these studies.

We encountered challenges in determining the methodologies used by these studies to mitigate confounding variables. This limitation is likely due to the relatively older nature of these studies, during which there was a tendency to place less emphasis on rigorously controlling for confounding factors.

SSI

**Figure 4:**
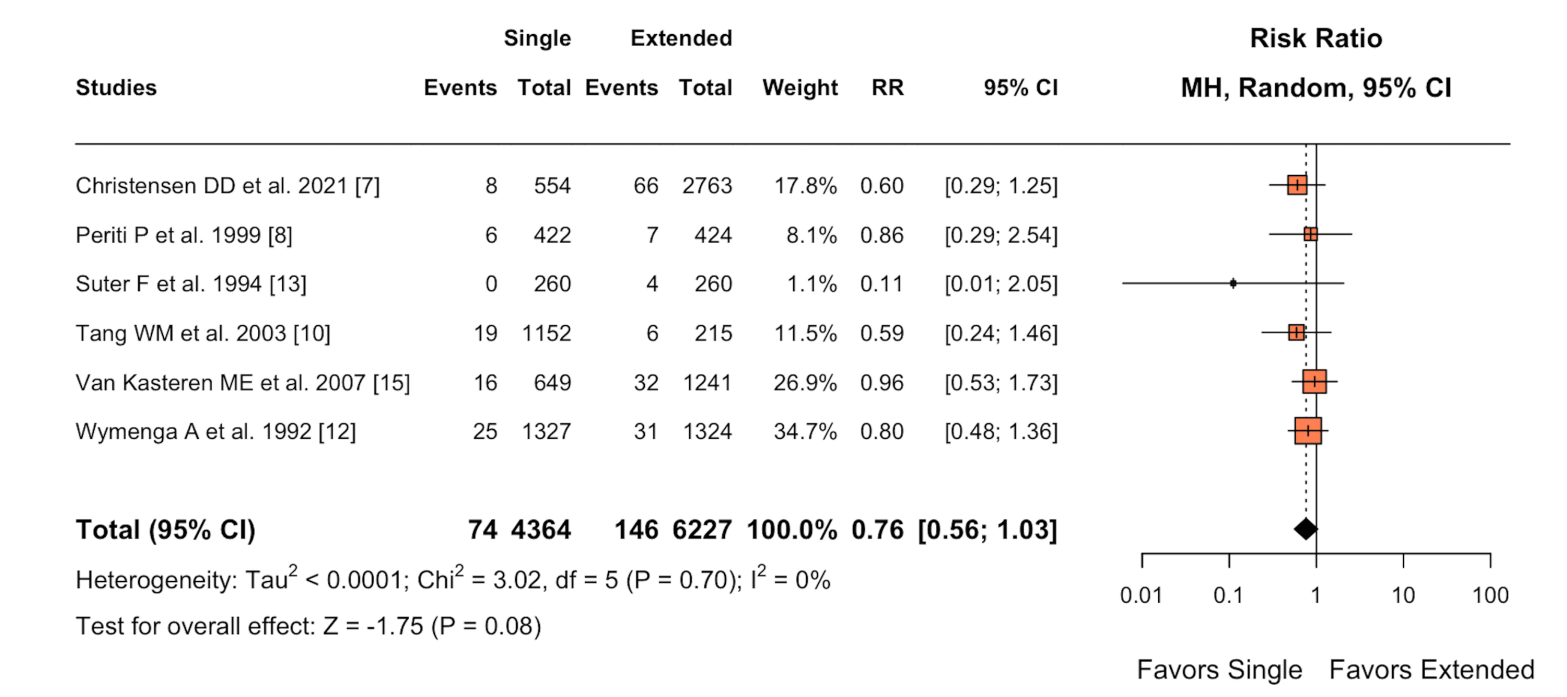
Forest plot of primary endpoint: SSIs SSI, surgical site infection

Patients who received a single dose of antibiotic prophylaxis showed a lower incidence of SSIs postoperatively compared to those who received extended antibiotic prophylaxis (RR 0.76; 95% CI 0.56-1.03; I² = 0%) (Figure 4). However, this difference was not statistically significant, with a p-value greater than 0.05 (P = 0.081).

PJI

**Figure 5:**
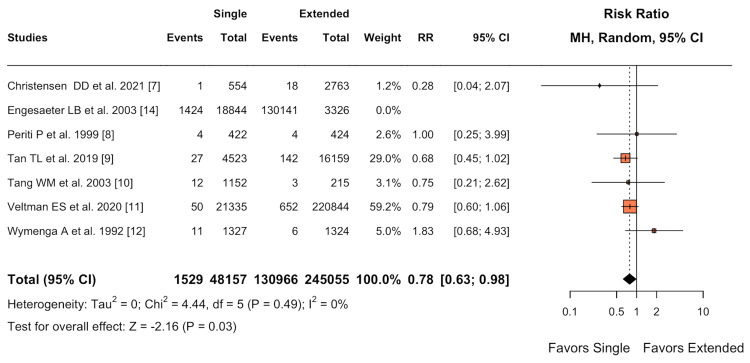
Forest plot of primary endpoint: PJIs PJI, periprosthetic joint infection

For the primary outcome measure of PJI, a statistically significant difference was found in favor of single-dose antibiotic prophylaxis (OR 0.78; 95% CI 0.63-0.98; I² = 0%) (Figure 5). This difference was statistically significant, with a p-value of 0.03, which is below the predetermined alpha level of 0.05.

Revision Surgery

**Figure 6:**
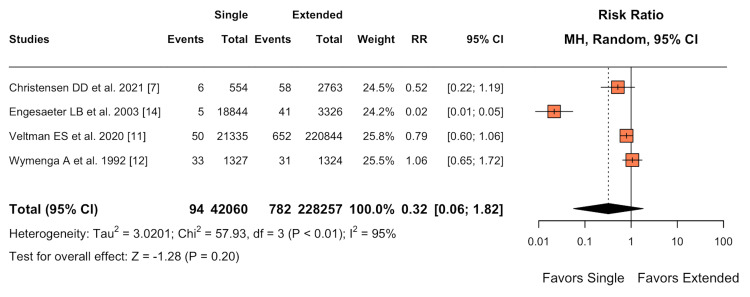
Forest plot of primary endpoint: revision surgery

**Figure 7:**
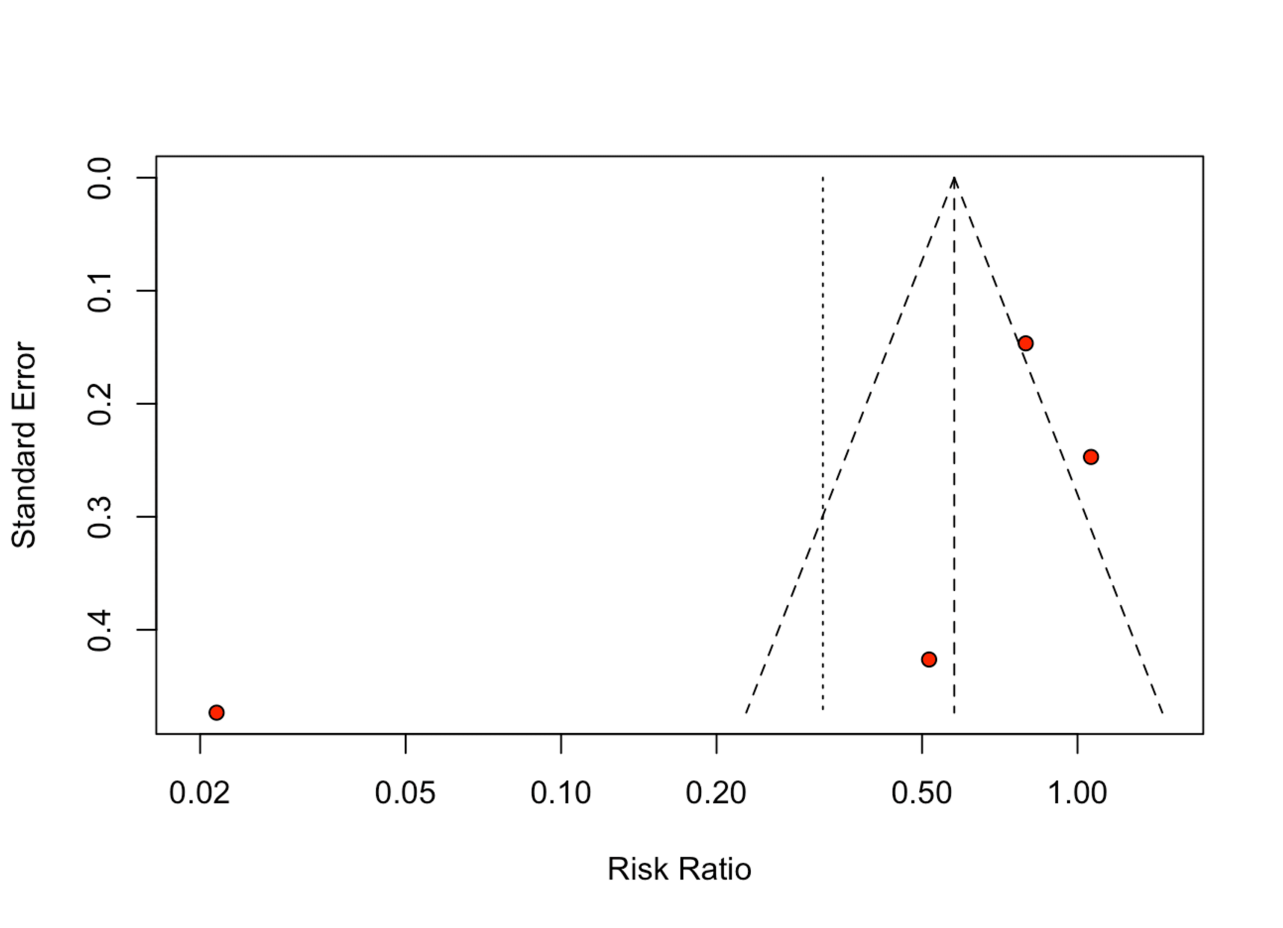
Funnel plot for primary endpoint: revision surgery

For the primary endpoint of revision surgery, no statistically significant difference was found between the single-dose antibiotic prophylaxis and extended antibiotic prophylaxis groups (RR 0.32; 95% CI 0.06-1.82; I² = 95%) (Figure 6). The p-value of 0.20 was greater than the threshold of 0.05, indicating no statistical significance. Notably, the high heterogeneity (I² = 95%) indicates substantial variability among the included studies, which is further discussed in the discussion section (Figure 7).

Discussion

This SRMA comprehensively evaluates the efficacy of single-dose versus extended antibiotic prophylaxis in primary hip and knee arthroplasty. Our findings suggest a potential shift in perioperative antibiotic administration protocols, with implications for clinical practice and healthcare resource allocation. The most notable result was the statistically significant reduction in PJI rates associated with single-dose antibiotic prophylaxis (OR 0.78; 95% CI 0.63-0.98; I² = 0%). This challenges the longstanding belief that extended antibiotic courses offer superior protection against postoperative infections. While the effect size was modest, it remains clinically relevant given the severe consequences of PJI and the high volume of arthroplasty procedures performed globally [16].

Several mechanistic hypotheses may explain the superiority of single-dose prophylaxis. Administering a single dose of antibiotics shortly before the surgical incision is favored for its simplicity and reduced risk of antibiotic resistance. This method is thought to offer adequate protection during the critical period of potential bacterial contamination while minimizing the risks associated with prolonged antibiotic use. It offers advantages such as lower cost, simplified administration, and decreased risk of antibiotic-related adverse effects. However, it may not provide adequate coverage for longer procedures or high-risk patients. Conversely, the extended dosing regimen, often lasting 24 hours or more post-surgery, aims to provide prolonged protection during the immediate postoperative period and may be more effective for high-risk patients or longer procedures. However, this approach increases the risk of antibiotic resistance, higher costs, more complex administration, and greater risk of antibiotic-related side effects (e.g., acute kidney injury, superimposed infections, and antibiotic resistance). Additionally, single-dose administration may achieve optimal tissue concentrations during the critical perioperative period, while extended regimens may result in subtherapeutic levels and increased risk of adverse effects [17].

Although the reduction in SSIs in the single-dose group did not reach statistical significance (RR 0.76; 95% CI 0.56-1.03; I² = 0%, P = 0.081), it aligns with the PJI findings. This consistency across infection-related outcomes strengthens the overall conclusion favoring single-dose prophylaxis. The lack of statistical significance may be due to insufficient statistical power for this outcome, highlighting the need for larger, well-designed studies focusing on SSIs as a primary endpoint.

Interestingly, there was no significant difference in revision surgery rates between the two prophylaxis strategies (RR 0.32; 95% CI 0.06-1.82; I² = 95%, P = 0.20). The high heterogeneity observed for this outcome suggests considerable variability in revision practices across studies, which could mask any true effect of antibiotic regimen on revision rates. This heterogeneity may arise from differences in surgical techniques, implant types, or institutional protocols for managing potential infections.

The methodological quality of the included studies varied, with RCTs generally demonstrating a lower risk of bias compared to non-RCTs. This discrepancy introduces a degree of uncertainty in interpreting our findings. The moderate to serious concerns identified in the bias assessment of non-RCTs emphasize the need for caution when generalizing these results to diverse clinical settings.

Several limitations of this meta-analysis should be considered. The heterogeneity in antibiotic protocols across studies - such as variations in drug choice, dosing, and timing - may complicate the interpretation of pooled results. Moreover, the inclusion of both RCTs and non-RCTs, while increasing the sample size and generalizability, introduces potential biases inherent to observational studies. Future research should prioritize large-scale, multicenter RCTs with standardized antibiotic protocols and comprehensive follow-up to address these limitations. We deemed it justifiable to conduct a new meta-analysis, given the substantial number of recent studies and the significant time since the last study by Ryan et al. [18].

Despite these limitations, the clinical implications of our findings are considerable. Adopting single-dose antibiotic prophylaxis could reduce antibiotic consumption, potentially mitigating the risk of antibiotic resistance and adverse drug reactions. Simplified prophylaxis protocols may also improve compliance and reduce healthcare costs associated with extended antibiotic administration.

## Conclusions

This meta-analysis supports the use of single-dose antibiotic prophylaxis in primary hip and knee arthroplasty, with observed reductions in PJI rates and trends toward lower SSI incidence. These findings suggest that single-dose regimens may offer a more favorable risk-benefit profile compared to extended protocols. However, limitations in study quality and heterogeneity call for further high-quality research to definitively establish the optimal antibiotic prophylaxis strategy in arthroplasty surgery. A significant limitation of this study is the unavailability of subgroup analysis due to insufficient data on secondary outcomes, which restricts our ability to draw nuanced conclusions about the efficacy of single-dose antibiotic prophylaxis in specific patient populations or surgical scenarios. The lack of detailed subgroup analysis also hampers our understanding of how factors such as patient demographics, comorbidities, or specific surgical techniques may influence the effectiveness of these regimens.

Future studies should address this limitation by prioritizing the collection of comprehensive data on secondary outcomes across diverse patient subgroups. This would enable more sophisticated analyses, potentially revealing variations in antibiotic efficacy among different populations and leading to more personalized and effective prophylaxis strategies. First and foremost, large-scale, multicenter RCTs employing standardized antibiotic protocols are needed to provide more robust and generalizable evidence, helping to establish best practices across healthcare settings. Extending follow-up periods in these trials is also crucial to allow for a more comprehensive assessment of late-onset infections, which may not be captured in shorter-term studies. Understanding the long-term implications of different prophylaxis regimens could significantly affect patient care and recovery. Additionally, conducting cost-effectiveness analyses comparing single-dose and extended prophylaxis regimens will provide valuable insights into the economic implications of these approaches, helping healthcare systems make informed decisions about resource allocation. Finally, future research should focus on identifying specific patient subgroups that may benefit more from extended prophylaxis, enabling more targeted and effective antibiotic use, potentially reducing overall antibiotic consumption while improving outcomes for high-risk patients. By addressing these areas, future studies can significantly enhance our understanding of antibiotic prophylaxis and contribute to the development of evidence-based guidelines for surgical procedures.

## Figures and Tables

**Figure 8:**
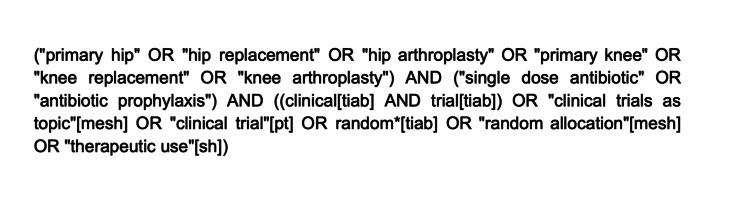
Search strategy

**Figure 9:**
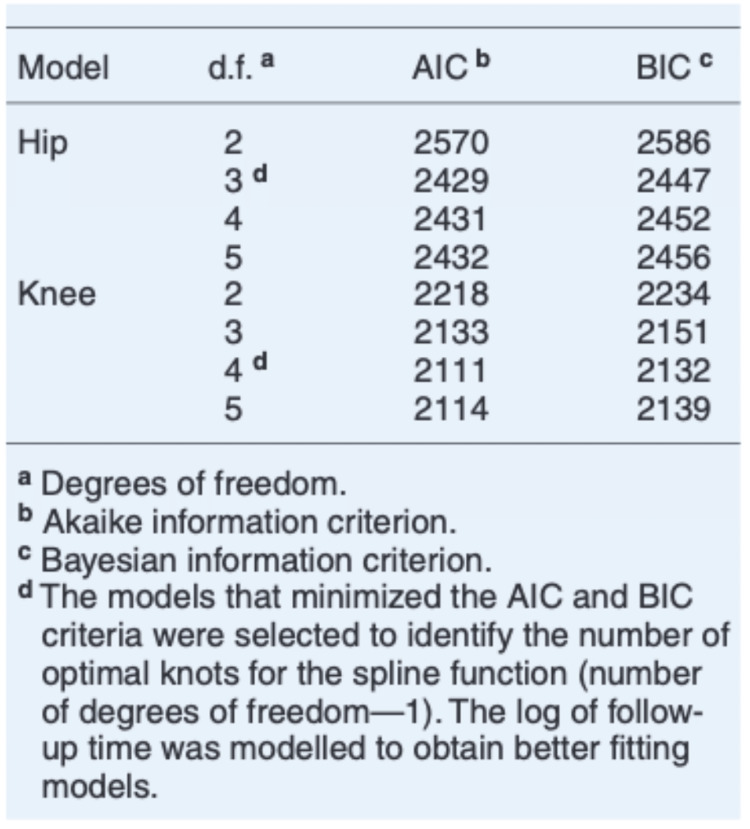
Appendix for Veltman et al. (2020) - person-years dataset ^a^ Degrees of freedom ^b^ Akaike information criterion ^c^ Bayesian information criterion ^d^ The models that minimized the AIC and BIC criteria were selected to identify the number of optimal knots of the spline function (number of degrees of freedom-1). The log of follow-up time was modeled to obtain better fitting models. Image source: Veltman et al. (2020) [11]; Creative Commons Attribution (CC BY) license

## Appendices

Appendix A

Appendix B
